# Theoretically designed interventions for colorectal cancer prevention: a case of the health belief model

**DOI:** 10.1186/s12909-020-02192-4

**Published:** 2020-08-17

**Authors:** Sakineh Rakhshanderou, Maryam Maghsoudloo, Ali Safari-Moradabadi, Mohtasham Ghaffari

**Affiliations:** 1 Environmental and Occupational Hazards Control Research Center, School of Public Health and Safety, Shahid Beheshti University of Medical Sciences, Tehran, Iran; 2 School of Public Health and Safety, Shahid Beheshti University of Medical Sciences, Tehran, Iran

**Keywords:** Educational intervention, Health belief model, Nutritional behavior, Colorectal cancer

## Abstract

**Background:**

According to the WHO, most chronic diseases, including cancer, can be prevented by identifying their risk factors such as unhealthy diet, smoking and physical inactivity. This research examined the effectiveness of a theory-based educational intervention on colorectal cancer-related preventive nutritional behaviors among a sample of organizational staff.

**Methods:**

In this interventional study, 110 employees of Shahid Beheshti University of Medical Sciences were randomly divided into two groups (intervention and control) with cluster sampling. The data gathering tool was a researcher-made questionnaire containing two parts of 10-dimensional information and health belief model constructs. The educational intervention was conducted for 1 month and in four sessions in the form of classroom lecture, pamphlet, educational text messages via mobile phones and educational pamphlets through the office automation system. Two groups were evaluated in two stages, pre-test and post-test. Data were analyzed using SPSS-18 software, analysis of Covariance (ANCOVA) and independent t-test (intergroup comparisons).

**Results:**

Two groups were evaluated for variables such as age, sex, education level and family history of colorectal cancer, and there was no significant difference between the two groups (*P* < 0.05). After the 2 months since intervention, except for the mean score of perceived barriers, which was not significant after intervention, the mean scores of knowledge, perceived susceptibility, perceived severity, perceived benefits, perceived self-efficacy, behavioral intention, and preventive behaviors were significantly increased after the intervention in the intervention group compared to the control group (*P* > 0.05).

**Conclusion:**

Implementation of educational intervention based on health belief model was effective for the personnel, and can enhance the preventative nutritional behaviors related to colorectal cancer.

## Background

**Fig. 1:**
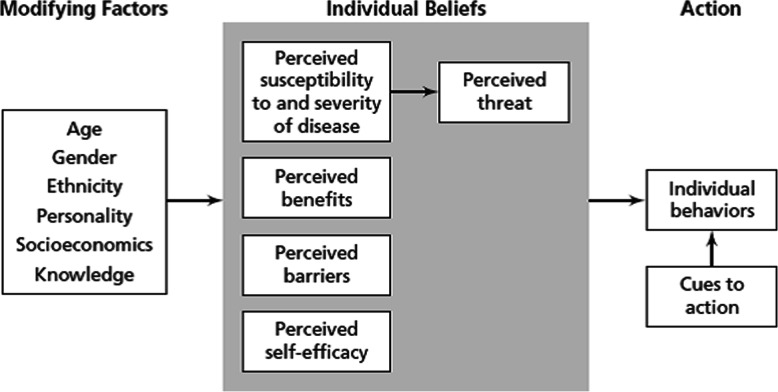
Health belief model’s components and links

Nearly 1.4 million new cases of colorectal cancer are diagnosed every year worldwide, with nearly half of the affected patients losing their lives due to the disease [1]. Approximately 4.6% of men (1 in 22) and 4.2% of women (1 in 24) are diagnosed with CRC during their life time [2]. The incidence of colorectal cancer in Iran ranges from 6 to 9.7 per 100,000 annually, with a death rate of about 1.198 per hundred thousand, and it accounts for approximately 13% of all gastrointestinal cancer-related deaths [3]. According to the latest cancer record in Iran, colon and rectum cancer ranked third in female cancers and fifth in male cancers. The global incidence of CRC is predicted to increase by 60%, to more than 2.2 million new cases leading to 1.1 million cancer deaths by 2030 [1]. The risk of colon cancer increases with age and is higher in men than in women [4]. Various factors are involved in the development of various types of cancer, including colorectal cancer, which can be attributed to genetic, environmental and dietary factors [5]. Among the risk factors of colorectal cancer, nutritional factors are considered to be the most important and preventable ones, so that 30 to 50% of cases can be prevented by proper nutrition [6, 7]. Colorectal cancer is also more common in Iran than in other Asian countries [8, 9]. Therefore, the need to educate people about the nutritional behaviors associated with colorectal cancer is becoming more and more evident. Theories and models identify factors that influence health and behavior – which means that they can be used to develop programs. The most effective training programs are based on the theory-driven approaches, which are rooted in behavior-changing models; also selecting appropriate model or theory is the first step in the process of planning a training program [10, 11]. As one of the most widely applied theories of health behavior, the Health Belief Model (HBM) posits that six constructs predict health behavior: perceived susceptibility, perceived severity, perceived benefits, perceived barriers, perceived self-efficacy and cues to action [12] (Fig. 1). The HBM posits that when an individual perceives a serious threat along with a way to reduce the threat they will be more likely to take action to reduce the threat [13]. The HBM has been applied to predict a wide variety of health-related behaviors such as being screened for the early detection of asymptomatic diseases [14]. The model has been applied to understand patients’ responses to symptoms of disease [14], lifestyle behaviors [10], and behaviors related to chronic illnesses [14], which may require long-term behavior maintenance in addition to initial behavior change [14]. The research hypotheses are: 1. an intervention based on the HBM can significantly promote colorectal cancer preventive behaviors. 2. The score for each and every construct of the HBM (e.g. perceived awareness and susceptibility, perceived severity, perceived benefits/barriers and perceived self-efficacy) is increased significantly after the intervention in the experimental group as compared to the control.

## Methods

### Study design and sampling

This interventional study was conducted at Shahid Beheshti University of Medical Sciences (Tehran, Iran) from October 2015 to June 2016.

In this study, using the sample size formula ($\mathrm{n}={\left({\mathrm{Z}}_{\frac{\propto }{2}}+{\mathrm{Z}}_{\upbeta}\right)}^2{\updelta}^2/\mathrm{d}2$) in which δ^2^ = 1.15, α = 1.96, β = 1.28, d = 0.5 and with an attrition rate of 10%, finally 110 women (55 subjects in the experimental and 55 in the control group) were considered. The random sampling method (clustering and simple random sampling) was used in this study. In order to choose from four faculties (faculties) of Shahid Beheshti University of Medical Sciences, four faculties were randomly selected and from these four faculties, two faculties were assigned as intervention group and 2 were considered as control group. Random sampling method was used to select samples from each cluster.

### Inclusion & Exclusion Criteria

Being under 50 years of age, having satisfaction to participate in the study, and not having serious diseases, including gastrointestinal diseases were the inclusion criteria. Also, not willing to continue with the study, not completing the questionnaire in full, and not attending in more than two educational sessions were the exclusion criteria.

### Measures

The researcher-made questionnaire was used for data collection in this study. Three sources of existed tools, literature review and expert view were used for item generation. This instrument consisted of two main parts as follow:

- **Part one:** Demographic questions about age, gender, educational level, and economic status.
- **Part two:** Constructs of the health belief model, which includes knowledge, perceived susceptibility, perceived severity, perceived benefits, perceived barriers, perceived self-efficacy, behavioral intention, and behavior (Table 1).

**Table 1:** Description of study instrument

***Construct***	***No. of Items (Format)***	***Scoring (Range)***
*1) Knowledge; refers to a theoretical or practical understanding of a subject*	11 items (true-false-don’t know)	‘Correct’ response = 2, ‘don’t know’ response = 1, ‘incorrect’ response = 0 (0–22)
*2) Perceived Susceptibility; refers to subjective assessment of risk of developing a health problem*	4 items/ 5-point Likert Scale (strongly disagree to strongly agree)	strongly disagree = 1, disagree = 2, no idea = 3, agree = 4, strongly agree = 5 (4–20)
*3) Perceived severity: Perceived severity refers to the subjective assessment of severity of a health problem and its potential consequences.*	6 items/5-point Likert Scale (strongly disagree to strongly agree)	strongly disagree = 1, disagree = 2, no idea = 3, agree = 4, strongly agree = 5 (6–30)
*4) Perceived benefits: Health-related behaviors are also influenced by the perceived benefits of taking an action.*	7 items/5-point Likert Scale (strongly disagree to strongly agree)	strongly disagree = 1, disagree = 2, no idea = 3, agree = 4, strongly agree = 5 (7–35)
*5) Perceived barriers: Health-related behaviors are also a function of perceived barriers to taking action.*	9 items/5 point Likert Scale (strongly disagree- strongly agree)	strongly disagree = 1, disagree = 2, no idea = 3, agree = 4, strongly agree = 5 (9–45)
*6) Perceived Self-efficacy: refers to an individual’s perception of his or her competence to successfully perform a behavior*	5 items/5 point Likert Scale (strongly disagree- strongly agree)	strongly disagree = 1, disagree = 2, no idea = 3, agree = 4, strongly agree = 5 (5–25)
*7) Behavioral intention; refers to a person’s perceived probability or “subjective probability” that he or she will engage in a given behavior.*	5 items/5-point Likert Scale (strongly disagree to strongly agree)	strongly disagree = 1, disagree = 2, no idea = 3, agree = 4, strongly agree = 5 (5–25)
*8) Behavior; refers preventative behaviors associated with colorectal cancer.*	5 items/5-point Likert Scale (Always to never)	always = 5, often = 4, sometimes = 3, rarely = 2, never = 1

### Validity and reliability

Face and content validities were applied for validation phase. Reliability was confirmed based on methods of test-retest and internal consistency (Cronbach’s alpha). For face validity, a survey was done on 4–5 employees about the difficulty in understanding the words and phrases, the probability of misunderstanding the phrases, and lack of clarity in the meaning of the words. Some modifications were made to the tool’s questions. To determine the content validity of the questionnaire, two gastroenterologists, five health education and health promotion specialists, and one related expert were asked to complete the questionnaire. The initial questionnaire had 52 questions. The constructs of knowledge, perceived susceptibility, perceived severity, perceived benefits, perceived barriers, perceived self-efficacy, intention and behavior had 11, 4, 6, 7, 9, 5, 5, and 5 questions respectively. Internal consistency was used to determine the reliability of HBM structures. The Cronbach’s alpha coefficient was 0.72 for all structures and was statistically acceptable. The re-test was used to ensure the reliability of the awareness variable. In this way, 15 employees completed the questionnaire twice and the ICC = 0.70 was obtained. Also, construct validity was performed by exploratory analysis method. The KMO value was 0.75 and Bartlett’s research showed the significant correlations among the items (*χ*2 = 1342.040, df = 435, *P* < 0.001); therefore, the data were suitable for conducting factor analysis.

### Intervention

Both intervention and control groups were pre-tested using the questionnaire. The analysis of educational needs determined the educational methods (educational package), and the number of educational sessions was obtained by the pre-test results. Assurance about readability, comprehensibility and not complexity of educational contents for participants was obtained by pre-testing materials (such as; pamphlets, messages, etc.) in a sample of 10 employees who were not included in main research.

#### Educational intervention based on educational text massages

Over the course of 10 days, ten text messages were sent to the employees in the intervention group at 8 am, most of which had been prepared according to the educational objectives of the constructs of knowledge, perceived susceptibility, perceived benefits, perceived barriers and perceived self-efficacy.

#### Educational pamphlets

Two pamphlets were given to the employees during two separate sessions, along with simultaneous provision of individual counseling. There was a possibility of questioning and answering any ambiguity regarding the content of pamphlets. The first pamphlet contained sections on the signs and symptoms of colorectal cancer and the risk factors of this cancer, and the second pamphlet contained sections on methods of preventing this cancer.

#### Educational packages in the office automation system

Educational packages were uploaded on the staff automation system for 10 days and the employees were asked to study it during the working hours.

**Fig. 2:**
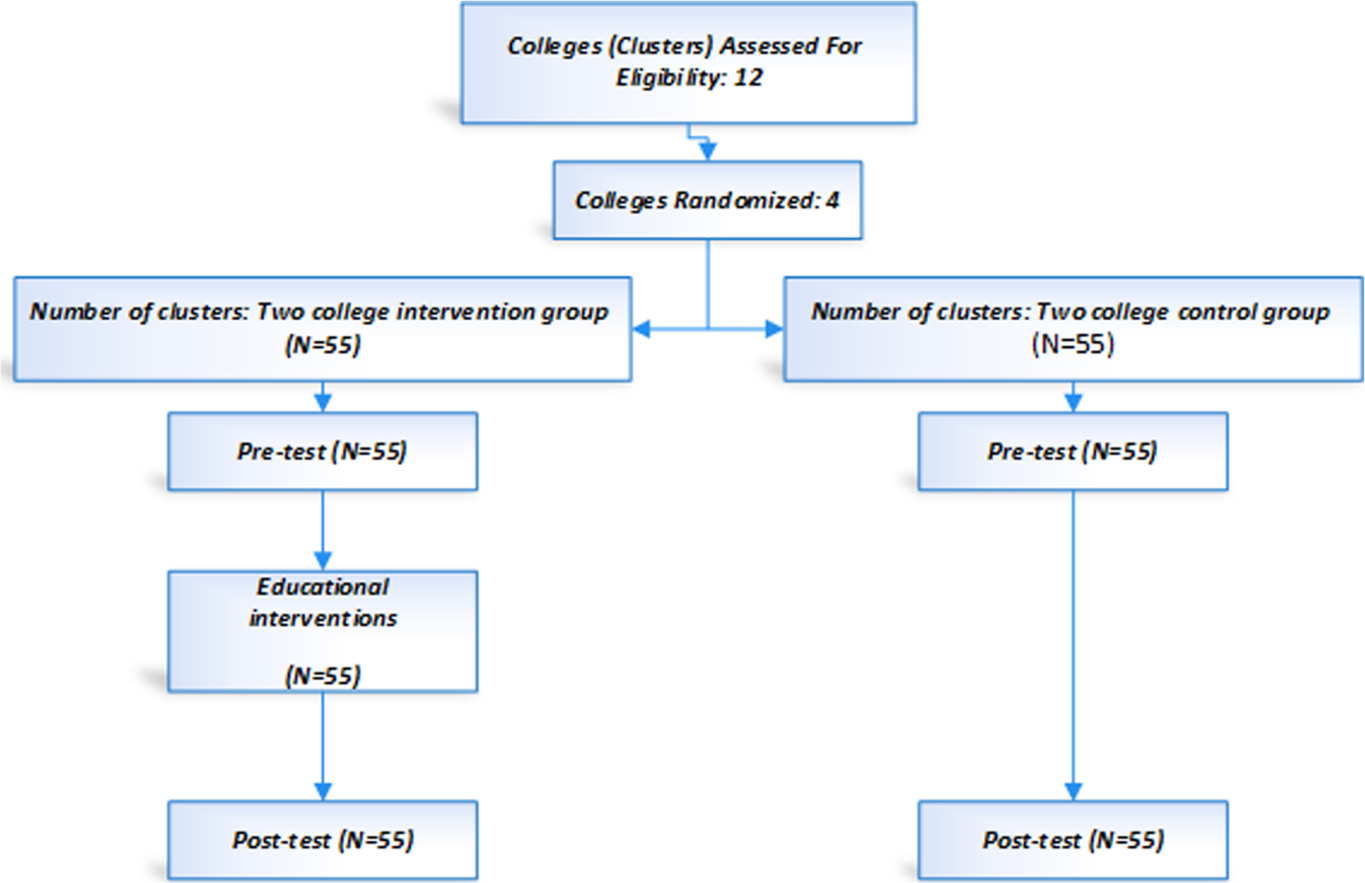
Schematic diagram of designed interventions for colorectal cancer prevention

The intervention was conducted 1 month and follow-up 2 months after the intervention. The educational contents were taken from the trusted sources of the Ministry of Health, complemented by what the staff needed to know about promoting nutritional behaviors related to the prevention of colorectal cancer. The education varied in form across the model constructs. For perceived susceptibility, the facts and figures of the incident rate of colorectal cancer were presented in the class, and for perceived severity, images of colorectal cancer problems were used. Also, for perceived barriers, educational materials were used to somehow incite the individuals to analyze the cost of optimal behavior against the costs of risks, time, etc. involved in unhealthy behavior. The educational content used for perceived benefits intended to raise awareness on the usefulness of health promoting behaviors to reduce the risk of illness or to understand the benefits of healthy behaviors. In Fig. 2, the research process is presented in general.

### Ethical considerations

At first, a permission was obtained from the university to conduct the study and attend the healthcare center. The samples were assured about the confidentiality of their specifications and information. They were also told that, their information will only be used for the purpose of this study and the data collection. The participants were allowed to enter and leave the study at any time. Suitable conditions were provided for a proper understanding of questions and responses for the subjects. After the end of the intervention period, the control group was also trained using the slides that were used to train the intervention group. An informed consent was obtained from the participants. The study on which these data analyses are based was approved by the Ethical Board Committee of Shahid Beheshti University of Medical Sciences.

### Data analysis

Data were analyzed by SPSS software. Kolmogorov Smirnov test was used to check the normality of the data. To assess the effectiveness of intervention on variables of knowledge, perceived susceptibility, perceived severity, perceived benefits, perceived barriers, perceived self-efficacy, behavioral intention and behavior in the intervention and control groups. Two groups were evaluated in two stages, pre-test and post-test. Data were analyzed using SPSS-18 software, analysis of Covariance (ANCOVA) and independent t-test (intergroup comparisons). In this study, the confidence level of 95% and the significance level of 0.05 were considered.

## Results

The findings of this study showed no drop out until the end of study. The questionnaire was completed in both groups in a complete and precise manner. Homogenization was done in the two groups by controlling variables such as age, sex, level of education, and related family history. The results showed no significant relationship within these variables (*P* < 0.05), (Table 2).

**Table 2:** Demographic and background variables in intervention and control groups before the intervention

Variable	***Group***	***Intervention group (N = 55)***	***Control group (N = 55)***	P –value*
		N (%)	N (%)	
**Age**	*25–35*	18(35.3)	18(36)	0.939
	*36–49*	32(62.7)	31(62)	
**Gender**	*Female*	16(31.4)	19(38)	0.484
	*Male*	35(86.6)	31(62)	
**Level of Education**	*Diploma*	5(9.8)	11(22)	0.138
	*Associate Degree.*	10(9.6)	5(10)	
	*Undergraduate degree and higher*	36(70.6)	34(68)	
**History of special diet compliance**	*Yes*	10(19.6)	9(18)	0.837
	*No*	40(78.4)	40(80)	
**Family history of cancer**	*Yes*	22(43.1)	21(42)	0.908
	*No*	29(56.9)	29(58)	

*Chi-square

**Fig. 3:**
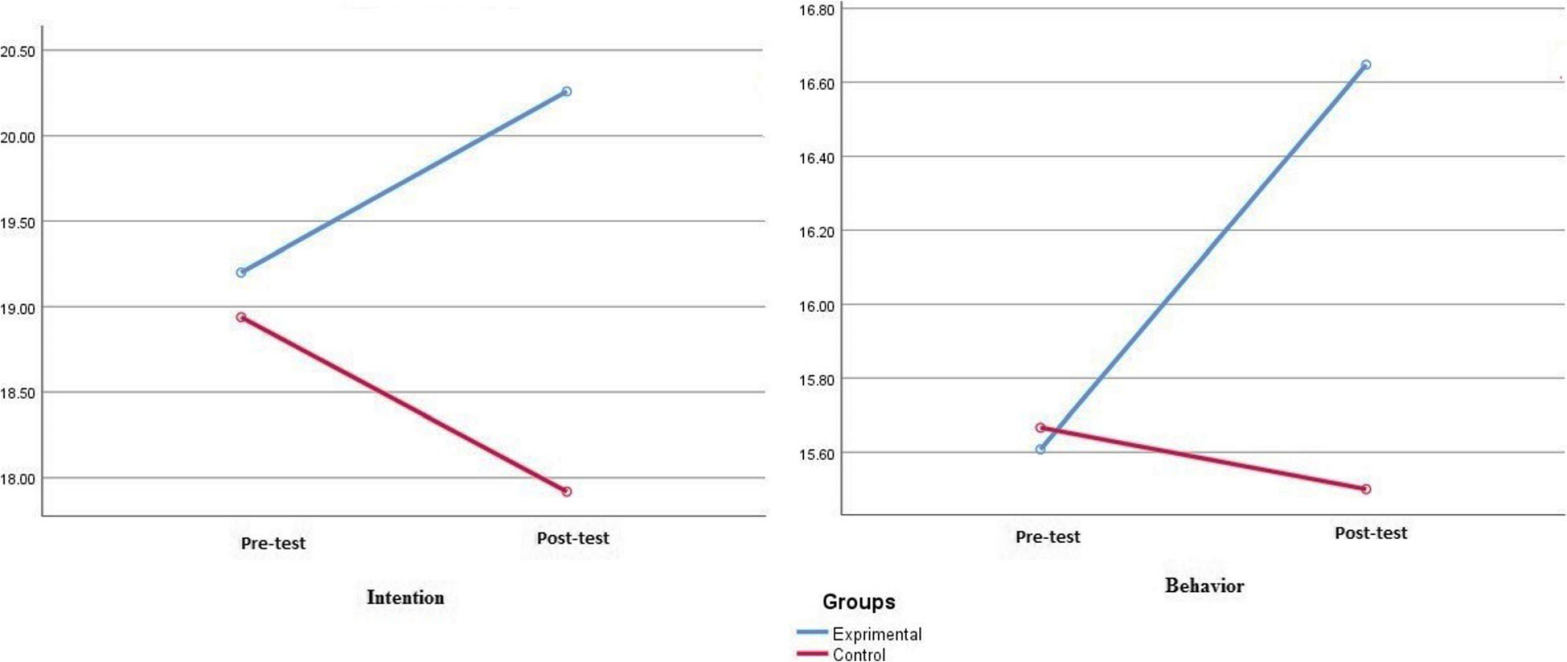
Mean scores of intention and behavior in the experimental and control groups before and after the intervention

Effectiveness of the educational intervention in improving knowledge, perceived susceptibility, perceived severity, perceived benefits, perceived self-efficacy, behavioral intention, and behavior, once age, gender and level of education factors were adjusted, was checked through ANCOVA. The results revealed that the intervention was successful in improving constructs of the Health belief Model significantly in participants (Table 3). The mean score of intention and behavior in the experimental and control groups before and after the intervention is presented in Fig. 3.

**Table 3:** Comparison of intervention and control groups in terms of health belief model constructs before and after the intervention

Constructs	Groups	Before intervention	After intervention	Mean Difference	*P* value^*^
		Mean ± SD	Mean ± SD		
Knowledge	Intervention	20.86 ± 4.49	26.23 ± 2.28	5.37 ± 2.21	< 0.001
	Control	19.57 ± 4.56	18.64 ± 4.70	−0.93 ± 0.14	
Perceived Susceptibility	Intervention	13.60 ± 3.70	15.58 ± 2.07	1.98 ± 1.63	< 0.001
	Control	11.35 ± 3.95	11.62 ± 3.41	0.27 ± 0.54	
Perceived Severity	Intervention	22.24 ± 4.72	24.18 ± 2.98	1.94 ± 1.29	< 0.001
	Control	20.93 ± 3.76	20.55 ± 3.08	−0.38 ± 0.68	
Perceived Benefits	Intervention	28.56 ± 3.75	30.35 ± 3.57	1.99 ± 0.18	< 0.001
	Control	27.77 ± 3.88	25.50 ± 4.23	−2.27 ± 0.35	
Perceived Barriers	Intervention	23.13 ± 5.57	22.11 ± 4.85	−1.02 ± 0.72	< 0.001
	Control	22.51 ± 4.10	24.00 ± 4.17	1.49 ± 0.07	
Perceived Self- Efficacy	Intervention	17.82 ± 3.39	20.03 ± 2.70	2.21 ± 0.69	< 0.001
	Control	16.50 ± 2.86	16.18 ± 3.05	−0.32 ± 0.19	
Behavioral Intention	Intervention	19.20 ± 3.06	20.26 ± 2.76	1.06 ± 0.3	< 0.001
	Control	18.93 ± 2.63	17.91 ± 2.99	−1.93 ± 0.36	
Behavior	Intervention	15.60 ± 1.68	16.64 ± 2.02	1.04 ± 0.34	< 0.001
	Control	15.66 ± 1.89	15.50 ± 1.73	−0.16 ± 0.16	

*Analysis of Covariance (ANCOVA)

## Discussion

The purpose of this study was to investigate the effects of educational interventions on the promotion of colorectal cancer prevention nutritional behaviors. The KMO (0.75) and Bartlett’s test (*P* < 0.001) results confirmed the suitability of the model for conducting factor analysis. The KMO is in the range 0–1. If the value of the inedex is near to one, the data are suitable for factor analysis. Kaiser (1977) at least KMO to 0. 60 determines [15]. Also, Bartlett test was used to confirm adequacy of the samples [16].

In the present study, the mean score of behavioral construct increased after the intervention in the intervention group, and there was significant difference between the two groups after the intervention in this regard. The results of this study are consistent with the findings of Abood et al. [17], Hart et al. [18], Roozitalabi et al. [19], Alidoosti et al. [20], and Davoodi et al. [21] studies. Behavioral intention is the thought of doing a behavior, and is considered as the immediate determinant of that behavior. The mean score in this construct as well increased in the intervention group after the intervention, and there was significant difference between the two groups after the intervention. In the study of Braun [22] and Gimeno et al. [23], the results were similar to the results of present study. Self-efficacy is a key prerequisite for behavior change. There was significant difference between mean score of perceived self-efficacy construct in the two groups after the intervention in this regard. The results of the study by Braun [22], Alidoosti et al. [20], and Hart et al. [18] are consistent with this finding. Perceived self-efficacy is considered as a strong motivational source and, in fact, is an indicator of the ability of individuals to organize themselves in pursuit of certain goals [24]. Studies show that individuals with a high level of perceived self-efficacy have a greater commitment to engage in activities at a time of challenges and difficulties, and spent more time and effort on such activities [25]. Such individuals are more likely to contribute to maintaining healthy behaviors and retrieve them, even after failure, and they have stronger intention and motivation. This not only improves the target adjustment, but also ensures achievement and sustainability in pursuit of the goals [26]. Another important factor is knowledge that can be pointed to its role in healthy behaviors. This study showed a significant difference in the two group in terms of the mean score of knowledge after the educational intervention. These results are consistent with the findings of Roozitalab [19], HO et al. [27] and Gimeno et al. [23] studies. Also, there was no significant difference in the control group before and after the intervention. Although increasing knowledge is an important step in changing attitudes and behaviors, it is not a major contributor to CRC prevention. Achieving the intention to behave is influenced by individual and environmental factors, so in addition to enhancing individual aspects, overcoming the structural and environmental barriers of the health system regarding the use of cancer prevention nutritional behaviors is also vital. In the present study, the mean score of perceived susceptibility and perceived severity constructs showed a significant difference between the intervention and control group after the educational intervention. Studies by Kolutek et al. [28], Wang et al. [29], Cengiz et al. [30] and Donadiki et al. [31] reported the role of beliefs regarding public health threats, perceived susceptibility and perceived severity in the health promotion behaviors. Becker et al. believed that one’s intention to self-care is influenced by his or her perception of vulnerability and the severity of disease outcomes [32]. Therefore, the need for interventions to increase the perception of society about the irreparable complications of diseases caused by unhealthy behaviors (Malnutrition habits) seems necessary. In this study, there was a significant difference between the two groups in terms of the constructs of perceived benefits after the educational intervention. This result is consistent with the findings of Grace et al. [33], Alidoosti et al. [20], and Abood et al. [17] studies. Also, in the present study, the mean score of perceived barrier construct decreased after the intervention. This was a good result, but it was not statistically significant. In the present study, the mean score of perceived barrier construct decreased after the intervention, which is not consistent with the results of studies by Moatari et al. [34], Grace et al. [18] and Gimeno et al. [23]. The study of Rajabi et al. (2000) identified some of the most important causes of barriers to nutrition in prevention of cancer [35], such as the difficulty of preventative measures, inappropriate economic status, and fear of cancer information. Therefore, strategies that overcome the individual and environmental barriers that affect nutritional behaviors should be addressed by planners and policymakers.

### Limitations

The limitations of this study, which could have had a relative effect on its findings, include the short duration of intervention, the sample size, the inability to follow the long term effect of the intervention, and the self-reporting of the subjects in responding to questions. However, the use of this method in such studies is inevitable and may lead to a bias of the “researcher-desired report”. In this study, anonymous questionnaire was used to minimize this bias.

## Conclusion

The findings of this study confirmed the effectiveness of health belief model-based education in improvement of colorectal cancer-related preventive behaviors. On the other hands, interventions based on HBM concepts could promote nutritional behaviors related to colorectal cancer prevention. Consequently, offering educational programs, including public information campaigns, workshops, videos, websites, exhibitions, etc. should be used to inform people about CRC symptoms and risk factors. Also, model-based education will have a greater effect on nutritional behaviors improvement by focusing on perceptions and enhancing beliefs about the applicability of the program and understanding the benefits and barriers.

## Data Availability

The datasets used and analyzed during the current study are available from the corresponding author on reasonable request.

## Abbreviations

CRC: Colorectal cancer
HBM: Health Belief Model
